# The effect of retirement on health behaviours: Evidence from Brazil

**DOI:** 10.1371/journal.pone.0338539

**Published:** 2025-12-16

**Authors:** Luan Vinicius Bernardelli, Wander Plassa, Michael Alexander Kortt, Michael B. Charles

**Affiliations:** 1 Special Academic Unit of Applied Social Sciences, Federal University of Goiás, Goiás, Goiás, Brazil; 2 Department of Economics, State University of Londrina, Londrina, Paraná, Brazil; 3 Department of Management Science and Engineering, Khalifa University, Abu Dhabi, United Arab Emirates; 4 Faculty of Business, Law and Arts, Southern Cross University, Gold Coast, Queensland, Australia; University of Zaragoza: Universidad de Zaragoza, SPAIN

## Abstract

**Objective:**

This study investigates the impact of retirement on health behaviours in Brazil in light of rising life expectancy and recent pension age reforms, focusing on how retirement affects well-being in a middle-income country.

**Methods:**

Using data from the 2013 and 2019 Brazilian National Health Surveys (PNS), this study analyses health behaviours among 54,741 individuals aged 50–80. Health behaviours (alcohol consumption, smoking, physical activity, sleep medication use, and diet) were measured using binary and continuous variables. Retirement status was defined as receiving a pension and not working, with Brazil’s minimum retirement age used as an instrumental variable to address endogeneity. Probit and IV probit models for binary outcomes and OLS and IV OLS models for continuous outcomes were estimated, with statistical tests supporting instrument strength and endogeneity.

**Results:**

The findings reveal a positive relationship between retirement and improvements in health behaviours. In the IV probit models, retirement is associated with increased physical exercise (β = 0.393, p < 0.05) and healthier eating habits (β = 0.371, p < 0.05). Men are less likely than women to reduce smoking. Retirement is linked to greater time spent engaging in physical exercise, reductions in alcohol consumption and smoking, together with healthier eating habits.

**Conclusion:**

These results have significant policy implications, underscoring the need to consider the potential long-term public health effects of increasing the retirement age, as it could result in higher public health burdens.

## Introduction

Life expectancy around the globe increased from 67 to 73 years from 2000 to 2019. Over the same period, life expectancy in Brazil reached 76 years, which is higher than the global average [1]. While this is a highly desirable outcome for Brazilians, a longer life expectancy naturally raises questions about the increased risk of chronic disease and disability. Against this background, numerous studies have examined the effect of retirement on health [2–7]. A greater life expectancy, however, necessitates more public expenditure with regard to health, social policies, social security, and other expenses [8,9]. In response to this emerging pressure on public sector budgets, policymakers in various countries have sought to prolong working lives, a policy that aims to provide greater tax revenue, a reduction in the state’s pension expenditure, and an increase in private savings, which can also support economic growth [10,11]. Such an outcome has mainly been observed in countries that have adopted a pay-as-you-go pension system [12]. Such initiatives might not be politically popular and can raise issues with employers [13,14], even if deemed fiscally expedient.

In short, pension reform proposals designed to improve public funding and sustainability must consider the effect of changes in life expectancy [15]. This is especially so given that a delayed retirement age, which *prima facie* might seem economically advantageous, might affect other outcomes, such as health and well-being [6,16]. In particular, increasing pension ages might paradoxically result in even more significant public costs, such as those associated with poor health, especially if retirement can ameliorate health outcomes for retirees. However, recent empirical evidence from Germany shows that raising the retirement age could have mixed effects: while it can prolong labour market participation, it also has the potential to increase unemployment, widen social disparities, and raise short-term health care costs among older workers [17,18].

Against this background, a recent and expanding literature has examined the causal effect of retirement on health behaviours [6,7,15]. Still, there is no consensus on the relationship between retirement and health [19–22]. Many reasons are attributed to this lack of consensus, including differences in methodological approaches, health measurements, retirement schemes, time spans, and the countries considered [5,12,23,24]. Regardless, there is a strong belief that increased leisure time and the possibility of self-improvement afforded by this greater leisure time can result in retirees devoting more attention to their overall well-being [6]. That said, some positive health behaviours require a degree of financial investment, such as gym memberships or eating fresh produce. This can be costly, especially considering the decreased income generally associated with retirement [6]. While some studies suggest that retirement is associated with improved health behaviours, such as increased physical activity or reduced smoking [3,25], others point to potential negative outcomes, including increased alcohol consumption [15,19]. This duality highlights the need for careful empirical analysis when assessing the consequences of retirement on health.

Importantly, the majority of research examining the relationship between retirement and health has focussed on developed countries like Australia [7,25,26], England [27,28], France [29], Germany [16,19], Switzerland [30], Japan [22,31], United States [3,21], the United Kingdom [4,32] and others [5,12,33]. Our study contributes to the literature by providing, to the best of our knowledge, the first results on whether retirement affects health behaviours in a Brazilian sample.

Brazil offers a distinctive context for examining retirement and health. Its public pension system comprises the General Social Security Regime (RGPS) for private-sector workers, the Pension Regime for Government Workers (RPPS) for civil servants, and a noncontributory benefit (BPC) for low-income elderly and disabled individuals [34,35]. The RGPS and RPPS operate largely on a pay-as-you-go basis [36,37], while the BPC is tax-funded [38]. Labour market informality means many workers face interrupted contribution histories, reducing access to contributory benefits [39,40]. During our study period (2013–2019), the statutory minimum retirement ages were 65 for men and 60 for women, before the 2019 reform raised women’s age to 62 [34,37,41–44]. Most pensions are modest, close to the minimum wage [45–48], and healthcare is provided through Brazil’s universal Unified Health System (SUS) [49–52]. Together, these features – a broad but modest pension system, universal healthcare, and frequent pension reforms – make Brazil a critical case for understanding how retirement influences health behaviours in a middle-income setting.

The objective of this study is to investigate the relationship between retirement and health behaviours in Brazil using nationally representative data from the 2013 and 2019 Brazilian National Health Surveys. We apply an instrumental variable approach exploiting statutory retirement age thresholds to address potential endogeneity and omitted variable bias.

## Materials and methods

### Data source

As stated above, the data used in this study were sourced from the 2013 and 2019 Brazilian National Health Surveys (*Pesquisa Nacional de Saúde* – PNS). These large and nationally representative surveys collect detailed information on health behaviours alongside key economic and social data from respondents [53,54]. The 2013 and 2019 Brazilian National Health Surveys received ethics approval from the National Research Ethics Committee [53,54]. The 2013 PNS collected data from 60,202 respondents, while the 2019 PNS collected data from 90,846 respondents. Since this study focuses on the effects of retirement on health behaviours, our analysis is restricted to respondents between the ages of 50 and 80, which is a comparable age range to that used in similar studies [14,15,21]. This range reflects Brazil’s institutional context, where early retirement pathways (e.g., contributory rules) and post-retirement labour force participation are common, resulting in a more gradual transition into retirement compared to countries with more rigid retirement patterns. To address potential concerns that including individuals in their early 50s or late 70s might dilute the estimated effects, we conducted robustness checks restricting the sample to narrower windows (55–75 and 60–70). The results were consistent with our main findings reported below, indicating that our conclusions are not sensitive to the choice of age window and that our definition effectively captures Brazil’s relevant retirement transition period.

**Fig 1:**
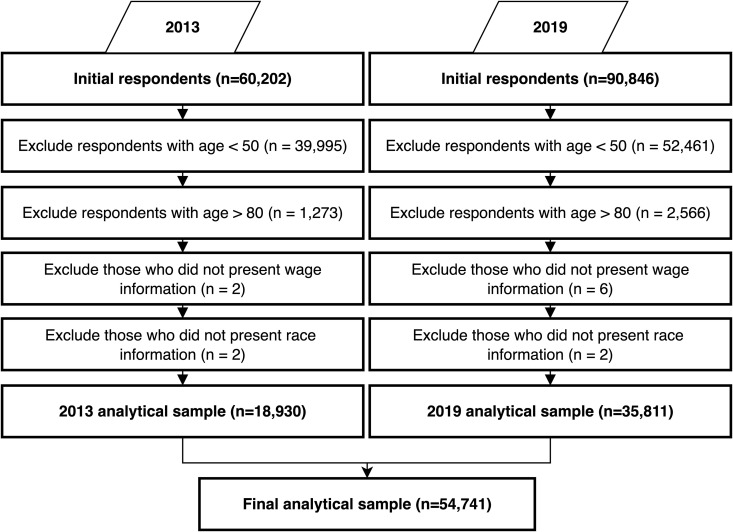
Flow diagram of data exclusion criteria.

The slightly higher proportion of women in our analytical sample likely reflects survey timing and labour force participation patterns, as older women are marginally more likely to be at home during interviews – a pattern commonly seen in household surveys [39,53,54]. Furthermore, a small proportion of cases were excluded owing to non-response (Fig 1). Thus, our final analytical sample was based on 18,930 and 35,811 respondents for 2013 and 2019, respectively (a total sample of 54,741 individuals).

### Dependent variables

Based on survey questions related to the frequency of alcohol consumption, smoking, participation in physical activity, taking sleep medication and fruit and vegetable consumption, we constructed five binary and five ‘continuous’ dependent variables (Table 1).

**Table 1:** Questions and definitions of main variables.

Dependents variables	Questions	Probit	OLS
Heavy drinkers	In general, on the day you drink, how many doses of alcoholic beverages do you consume?^a^	1 = number the doses ≥ 5; 0 = otherwise	Number of doses
Smoker	On average, how many industrialised cigarettes do you currently smoke per day?	1 if number the cigarettes ≥ 1; 0 = otherwise	Number of cigarettes
Physical exercise^b^	In general, on the day you practise physical exercise or sport, how long does this activity last? How many days a week do you usually (used to) practise physical exercise or sport?	1 = practiced ≥ 150 minutes of physical exercise per week; 0 = otherwise	Total minutes of physical activity per week
Sleep medicine	In the last two weeks, how many days did you use the medication to help you sleep?	1 = if number of days taking medication ≥ 1; 0 = otherwise	Number of days
Healthy diet^c^	1a. How many days of the week do you typically consume cooked vegetables, such as kale, carrots, chayote, eggplant, and zucchini (excluding potatoes, cassava, or yams)? 2a. On how many days of the week do you usually eat fruit?	1 = if eats vegetables and fruit more than four days per week; 0 = otherwise	Number of days per week eating vegetables and fruit
**Independent variable**	**Question**	**Classification**	
Retirement^d^	In the survey month, did you receive any retirement or pension benefits?	1 = retired; 0 = otherwise	

^a^A standard alcoholic drink is equivalent to one can of beer, one glass of wine, or one shot of *cachaça*, whiskey, or any other distilled alcoholic beverage.

^b^This threshold reflects international recommendations for minimum weekly physical activity to promote health and prevent noncommunicable diseases.

^c^This variable has been calculated as the sum of the number of days of eating vegetables and fruit so that the value can range from 0 to 14.

^d^For those who indicated working at least one hour in any paid cash activity in the survey month, retirement status was replaced with zero (if, initially, it was classified as 1).

The binary variables were used to classify respondents engaged in heavy alcohol consumption, defined as individuals who typically consume five or more alcoholic beverages on a single occasion (yes/no), smoked tobacco (yes/no), engaged in physical exercise in accordance with the World Health Organization’s recommendation (yes/no), took sleep medication (yes/no), and consumed fruit and vegetables more than four days per week (yes/no). The ‘continuous’ dependent variables were designed to capture the frequency of alcohol consumption (number of doses consumed in a sitting), smoking (number of cigarettes smoked per day), physical exercise (number of minutes per session), using sleep medication (number of days in the last two weeks), and fruit and vegetable consumption (number of days per week).

### Independent variable

In this study, retirement is our principal independent variable of interest. Survey respondents were classified as being retired if they received a retirement income and did not engage in paid work, which is consistent with the approach of similar studies [26,55]. However, when examining the relationship between retirement and its attendant impact on health behaviours, the endogeneity problem arises. For instance, while retirement (R) may influence health behaviours (HB) (i.e., R → HB), it is also possible that health behaviours could influence retirement (i.e., HB → R). For example, individuals who engage in persistent unhealthy behaviours, such as excessive alcohol consumption or physical inactivity, may develop health conditions that lead to an early exit from the labour market. In this scenario, health behaviour precedes and potentially drives retirement. This reverse causality underscores the importance of employing an instrumental variable strategy to isolate the causal effect of retirement on health behaviours.

Therefore, a suitable instrument is required to deal with the endogeneity problem. Related studies deal with this issue by using policy-determined retirement age as an instrumental variable for retirement [4,6,56–58]. As a result, we used Brazil’s minimum retirement age (i.e., eligibility to receive a government pension) as a binary instrumental variable for retirement. The instrumental variable was coded as ‘1’ if the respondent had reached the minimum retirement age during 2013–2019 (i.e., 60 for women and 65 for men, before the 2019 reforms) and coded as ‘0’ otherwise [59]. As rural workers in Brazil are subject to different retirement rules, with earlier statutory ages (typically 55 for women and 60 for men), we conducted a sensitivity analysis excluding individuals residing in rural areas from the sample. Although this proxy does not fully capture employment-based classification, it helps to mitigate concerns regarding differential eligibility rules. The models’ results remained robust, suggesting that rural-specific retirement thresholds do not drive our main findings.

While the relevance of our instrument is empirically supported through strong first-stage results, the exogeneity assumption relies on institutional features of Brazil’s retirement system. The minimum retirement age is a binding eligibility criterion for accessing pension income, which is the primary source of income for most older adults in Brazil. Given this institutional setup, it is plausible to assume that reaching the minimum retirement age affects health behaviours primarily through the induced change in retirement status. Although anticipatory behavioural changes are possible, their scope is likely limited by the conditional nature of pension eligibility (which also requires contribution time) and the economic necessity of continued work for many individuals before retirement. If some anticipatory behavioural changes occur, the resulting bias could in principle go in either direction – depending on whether pre-retirement adjustments improve or worsen health – and is likely modest and concentrated in a narrow window before eligibility. We therefore note anticipation as a limitation rather than assuming it makes the IV estimates conservatively biased. In addition, because the PNS is cross-sectional, we cannot implement event-study tests of pre-trends; accordingly, we interpret the IV estimates as LATEs for compliers induced by the age thresholds. Furthermore, although Brazil underwent pension reforms in 2019, these did not retroactively alter the statutory ages relevant to our sample during the period 2013–2019. For instance, if retirement status is measured with some degree of error, such as respondents misreporting whether they are fully or partially retired, the estimated coefficients in the Probit models would tend to be biased toward zero. In this case, the relationship between retirement and health behaviours would appear weaker than it truly is. Using an instrumental variable corrects for this attenuation, which can help explain why the IV estimates are larger in magnitude than the corresponding Probit estimates.

### Control variables

The control variables included in the subsequent statistical analysis were income, education, biological sex, religion, marital status, whether the respondent resided in a rural area, and geographical region. In addition, we included age and age squared in all models to flexibly capture potential non-linear effects of ageing on health behaviours and address age-related confounding. Income was divided into three categories (high/medium/low), with low-income earners chosen as the excluded reference group. For our education variable, we consider the highest level of education achieved based on the nine-year elementary education system. More specifically, we consider people with no education or incomplete elementary school or equivalent as belonging to the first level of education. Our second level of education comprises people who completed primary school or partially completed secondary school. Our third level of education comprises those who have completed secondary school. Finally, our highest level includes people with some higher education. Therefore, educational attainment was divided into four categories (0–8 years; 9–11 years; 12 years; 13 + years), with those respondents in the lowest educational attainment category being selected as the reference group. Biological sex was divided into two categories (male/female), with those identifying as female being chosen as the reference group. Religious attendance was split into two categories: those who attended religious activities at least once a week in the last twelve months and those who did not. Marital status was divided into two categories (1 = married; 0 = otherwise), and a binary variable was created for those respondents living in rural areas (1 = resides in a rural area; 0 = otherwise). We also included categorical variables to control for geographical regions (Northeast, North, Southeast, South, and Midwest) and an indicator variable for the survey year (1 = 2013; 0 = 2019).

### Statistical analysis

After reporting our summary statistics, we estimated a series of regression models to examine the association between retirement and health behaviours:


$$HB=\alpha_{\text{0}}+\beta_{\text{1}}R+\beta_{\text{2}}X+\mu$$ (1)

In equation (1), HB is one of our five binary or five ‘continuous’ measures of health behaviours (i.e., drinking, smoking, physical activity, sleep medication, and healthy diet), R is our retirement variable (1 = retired; 0 = otherwise), X is our vector control variable as is described above, and µ is our error term. To deal with the endogeneity problem, we used Brazil’s minimum retirement age as an instrumental variable for retirement (1 = the respondent had reached the minimum retirement age; 0 = otherwise):


$$R=\alpha_{\text{0}}+Retirement\_Age+\beta_{\text{2}}X+\mu$$ (2)

Our empirical analysis formally tested whether our theoretically endogenous variable – retirement – can be treated as exogenous in our models (i.e., the null hypothesis is that our retirement variable is exogenous). Tests were also conducted to ensure that our chosen instrument – i.e., policy-determined retirement age – meets the necessary statistical criteria. Following similar studies [6,15,25,60–62], we calculated the F-statistic on the excluded instrument to test whether we had a weak instrument. Values above 10 suggest that the instrument is acceptable (i.e., not weak) [63]. We also calculated the Kleibergen-Paap Wald rk F and found that the corresponding values were greater than the critical values [63]. In addition, the full first-stage regression results are presented in Table 2 and provide a more comprehensive view of the relationship between the instrument and the endogenous variable.

**Table 2:** First-stage regression estimates: instrumenting retirement with minimum eligibility age.

	IV Probit (Table 4)	IV Probit (Table 5) (Panel A – men)	IV Probit (Table 5) (Panel B – women)	IV REG (Table 7)
	*β (SE)*	*β (SE)*	*β (SE)*	*β (SE)*
Age Retirement	0.127***	0.161***	0.065***	0.1269***
	(0.007)	(0.012)	(0.011)	(0.0074)
Constant	−2.209***	−1.658***	−3.427***	−2.209***
	(0.105)	(0.148)	(0.169)	(0.1054)
Controls	Yes	Yes	Yes	Yes
Observations	54,741	24,661	30,080	54,741

* p < 0.1, ** p < 0.05, *** p < 0.01.

Notes: Each column reports the first-stage coefficient from retirement regressions on the statutory-age eligibility indicator. Columns are aligned with the outcome specifications in Tables 4, 5 and 7. The control variables included in the first stage regression estimates are the same as those reported in Table 3.

These first-stage estimates confirm that reaching the statutory minimum retirement age strongly predicts retirement status, reinforcing the relevance assumption central to our instrumental variable approach. In summary, our statistical tests did not uncover a weak instrument problem.

To improve transparency, we distinguish between tests of instrument relevance and tests of exogeneity. Instrument relevance is assessed using the Kleibergen-Paap rk Wald F-statistic, which evaluates whether the excluded instrument (minimum retirement age) is sufficiently correlated with the endogenous regressor (retirement). A value above 10 suggests that the instrument is not weak. Exogeneity is tested using Wald tests of exogeneity in the IV Probit models and robust Hausman-type tests (via endogtest) in the IV OLS models. These tests evaluate whether the correlation between the endogenous regressor and the error term is statistically significant. Throughout the manuscript and tables, we refer to these explicitly as “Exogeneity tests” to clarify their purpose and interpretation. The Wald test evaluates the null hypothesis that retirement is exogenous (i.e., uncorrelated with the error term). A rejection of the null (*p* < 0.10) supports the presence of endogeneity and justifies using the instrumental variable approach, whereas failure to reject it indicates no statistical evidence of endogeneity. The degree of endogeneity may vary across models depending on the outcome variable considered, as unobserved determinants of behaviours such as smoking, drinking, or exercising may correlate differently with retirement. To maintain comparability across results, we report both the baseline (OLS/Probit) and IV (IV-OLS/IV-Probit) estimates, emphasising the IV results when endogeneity cannot be rejected. A concise description of the test and its interpretation appears in the regression table notes.

Thus, to analyse comprehensively the relationship between retirement and health behaviours, we estimated (i) a series of probit and IV probit models using our five binary health behaviour dependent variables and (ii) a series of OLS and IV OLS models using our five ‘continuous’ health behaviour dependent variables.

It is important to note that the main estimations were performed without sampling weights. However, to assess the robustness of our results, we re-estimated the main models using the official sampling weights, and the results were very similar to those reported in the main tables, indicating that the findings are not sensitive to the inclusion of sampling weights.

Given the instrumental variable design, the estimates presented in this study should be interpreted as Local Average Treatment Effects (LATEs). Specifically, our results reflect the causal impact of retirement on health behaviours among individuals whose retirement status is influenced by reaching the minimum retirement age – a relevant group in Brazil, where many individuals retire precisely when they become eligible for pension benefits. This interpretation is aligned with previous empirical studies using similar designs [7,18,27].

### Ethics approval

This study used publicly available secondary data from de-identified existing unit records from the 2013 and 2019 Brazilian National Health Surveys. The National Research Ethics Committee approved this survey in July 2013 and August 2019. (CAAE 10853812.7.0000.0008) and (CAAE 11713319.7.0000.0008). The survey data is publicly available and was downloaded by the researchers from the *Instituto Brasileiro de Geografia e Estatística* website (https://www.ibge.gov.br/en/statistics/social/justice-and-security/16840-national-survey-of-health.html?edicao=19375).

## Results

### Descriptive statistics

Table 3 presents the variables, their respective definitions, and sample means derived from the 2013 survey, the 2019 survey, and the pooled means obtained by combining data from both surveys. Our pooled sample was comprised of 45% men and 55% women. The mean age was 61.84 years, and nearly half of the respondents reported being married (47%). A majority of respondents identified as being non-white (58%), and 22% of respondents reported that they resided in a rural area. Regarding geographical distribution, 32% of survey respondents indicated they reside in the Northeast of Brazil, with 16% in the North, 26% in the Southeast, 14% in the South, and 12% in the Midwest.

**Table 3:** Sample characteristics.

Variables	Definitions	Mean (2013)	Mean (2019)	Mean (Pooled)
**Dependent variables**				
***Binary***				
Heavy drinker	1 = Heavy alcohol drinker 0 = otherwise.	0.05	0.08	0.07
Smoker	1 = smoker; 0 = otherwise.	0.12	0.09	0.10
Physical exercise	1 = practised ≥150 minutes of physical activity per week; 0 = otherwise	0.14	0.20	0.18
Sleep medicine	1 = took sleep medicine; 0 = otherwise.	0.12	0.12	0.12
Healthy diet	1 = consumed fruit and vegetables; 0 = otherwise.	0.23	0.37	0.32
***Continuous***				
Drinks	Number of doses of alcoholic beverages consumed.	0.73	1.14	1.00
Cigarettes Smoked	Number of industrialised or straw cigarettes smoked.	1.61	1.20	1.34
Physical exercise	Total minutes of physical activity per week	55.85	76.23	69.18
Sleep medication	Number of days sleep medication was taken.	1.17	1.19	1.18
Healthy diet	Number of days eating vegetables and fruit.	7.8	9.11	8.44
**Independent variables**				
Retired	1 = Received retirement income and does not work in any paid activity; 0 = otherwise.	0.41	0.42	0.41
***Controls***				
Age	Age in years	61.38	62.09	61.84
Age squared	Age squared	3,834	3,921	3,891
*Income*				
Low income *(ref.)*	1 = income less than the minimum wage; 0 = otherwise.	0.36	0.47	0.43
Medium income	1 = income between 1 and 3 times greater than the minimum wage; 0 = otherwise.	0.48	0.38	0.41
High income	1 = income over 3 times the minimum wage; 0 = otherwise.	0.16	0.15	0.15
*Education*				
0–8 years *(ref.)*	1 = yes; 0 = otherwise.	0.60	0.57	0.58
9–11 years	1 = yes; 0 = otherwise.	0.11	0.11	0.11
12 years	1 = yes; 0 = otherwise.	0.16	0.18	0.17
13 + years	1 = yes; 0 = otherwise.	0.13	0.15	0.14
Biological sex	1 = Male; 0 = female.	0.43	0.46	0.45
Religious attendance	1 = Attended religious activities at least once a week; 0 = otherwise.	0.40	0.40	0.40
Married	1 = Married; 0 = otherwise.	0.47	0.46	0.47
Non-white	1 = Non-white; 0 = otherwise.	0.55	0.59	0.58
Rural resident	1 = Resident of rural area; 0 = otherwise.	0.19	0.23	0.22
Northeast resident *(ref.)*	1 = Resident in Northeast of Brazil; 0 = otherwise.	0.30	0.34	0.32
North resident	1 = Resident in North of Brazil; 0 = otherwise.	0.16	0.16	0.16
Southeast resident	1 = Resident in Southeast of Brazil; 0 = otherwise.	0.27	0.25	0.26
South resident	1 = Resident in South of Brazil; 0 = otherwise.	0.14	0.14	0.14
Midwest	1 = Resident in Midwest of Brazil; 0 = otherwise.	0.12	0.11	0.12
**Instrument**				
Age retirement	1 = the respondent had reached the minimum retirement age; 0 = otherwise.	0.45	0.47	0.46
**Observations**		18,930	35,811	54,741

Notes: In 2019, the monthly minimum wage was R$998; in 2013, it was R$678.

Turning to our health behaviour variables, we find that 7% of respondents could be classified as heavy alcohol drinkers, defined as individuals who reported consuming five or more alcoholic drinks on a single occasion. Across the whole sample – including drinkers and non-drinkers – the average number of alcoholic doses consumed on drinking days was 1. In a similar vein, 10% of respondents were classified as smokers, and the mean number of cigarettes smoked per day was 1.34. In our pooled sample, 18% of survey participants met the recommended threshold of at least 150 minutes of physical activity per week, based on self-reported data, with an average exercise time of 69 minutes per week. Concerning sleep medication, 12% of our pooled sample reported taking sleep medication in the last two weeks, with an average duration of 1.18 days for the use of this medication. Finally, 32% of survey respondents reported consuming fruit and vegetables more than four days per week. Table 4 presents the findings obtained from our probit regression analysis. Here, we delve into the relationship between retirement and health behaviours. Importantly, in three out of five regressions, we observed no evidence of endogeneity, particularly pertaining to individuals’ drinking habits, physical exercise, and the utilisation of sleep medication.

**Table 4:** Probit regressions.

	Heavy Drinker	Smokers	Physical Exercise	Sleep medication	Healthy Diet
	*β (SE)*	*β (SE)*	*β (SE)*	*β (SE)*	*β (SE)*
Retirement	−0.153***	−0.074***	0.138***	0.230***	0.018
	(0.023)	(0.019)	(0.016)	(0.018)	(0.014)
Age	0.035*	0.108***	0.056***	−0.026*	0.032***
	(0.020)	(0.016)	(0.013)	(0.014)	(0.011)
Age squared	−0.001***	−0.001***	−0.001***	0.000*	−0.000*
	(0.000)	(0.000)	(0.000)	(0.000)	(0.000)
Medium income	0.119***	−0.028	0.168***	−0.010	0.270***
	(0.021)	(0.018)	(0.016)	(0.017)	(0.014)
High income	0.212***	−0.069**	0.442***	0.044*	0.517***
	(0.033)	(0.029)	(0.022)	(0.026)	(0.021)
Educ (9–11 years)	0.114***	0.058**	0.226***	−0.052**	0.187***
	(0.030)	(0.025)	(0.022)	(0.024)	(0.020)
Educ (12 years)	0.200***	−0.046**	0.355***	−0.114***	0.305***
	(0.025)	(0.023)	(0.019)	(0.022)	(0.017)
Educ (13 + years)	0.081**	−0.143***	0.602***	−0.109***	0.448***
	(0.033)	(0.029)	(0.022)	(0.026)	(0.021)
Biological sex (male)	0.800***	0.184***	0.052***	−0.457***	−0.314***
	(0.020)	(0.016)	(0.014)	(0.016)	(0.013)
Married	−0.211***	−0.272***	0.055***	0.011	0.092***
	(0.018)	(0.016)	(0.014)	(0.015)	(0.012)
Non-white	0.141***	0.043**	−0.020	−0.120***	−0.112***
	(0.021)	(0.017)	(0.015)	(0.016)	(0.013)
Religious attendance	−0.452***	−0.423***	0.246***	−0.011	0.191***
	(0.021)	(0.017)	(0.014)	(0.015)	(0.012)
Rural resident	−0.208***	−0.301***	−0.376***	−0.131***	−0.215***
	(0.024)	(0.022)	(0.020)	(0.020)	(0.016)
North resident	0.010	−0.093***	−0.174***	−0.290***	−0.148***
	(0.025)	(0.026)	(0.021)	(0.025)	(0.019)
Southeast resident	−0.210***	0.253***	−0.131***	0.113***	0.190***
	(0.025)	(0.021)	(0.018)	(0.019)	(0.016)
South resident	−0.535***	0.326***	−0.140***	0.130***	0.285***
	(0.036)	(0.025)	(0.022)	(0.023)	(0.020)
Midwest resident	−0.155***	0.137***	−0.050**	−0.020	0.099***
	(0.031)	(0.027)	(0.022)	(0.025)	(0.020)
2013	−0.259***	0.145***	−0.254***	−0.063***	−0.510***
	(0.020)	(0.016)	(0.014)	(0.015)	(0.013)
Constant	−1.747***	−3.921***	−2.616***	−0.128	−1.933***
	(0.611)	(0.501)	(0.410)	(0.434)	(0.360)
Exogeneity test	0.26	0.02	0.18	0.56	0.04
Endogeneity?	No	Yes	No	No	Yes
Observations	54,741	54,741	54,741	54,741	54,741

* p < 0.1, ** p < 0.05, *** p < 0.01.

Notes: The ‘Exogeneity test’ reports the p-value from the Wald test of exogeneity or a robust Hausman-type test. A p-value below 0.10 indicates evidence of endogeneity, justifying instrumental variable estimation; a p-value above 0.10 suggests no evidence of endogeneity, allowing the variable to be treated as exogenous.

**Fig 2:**
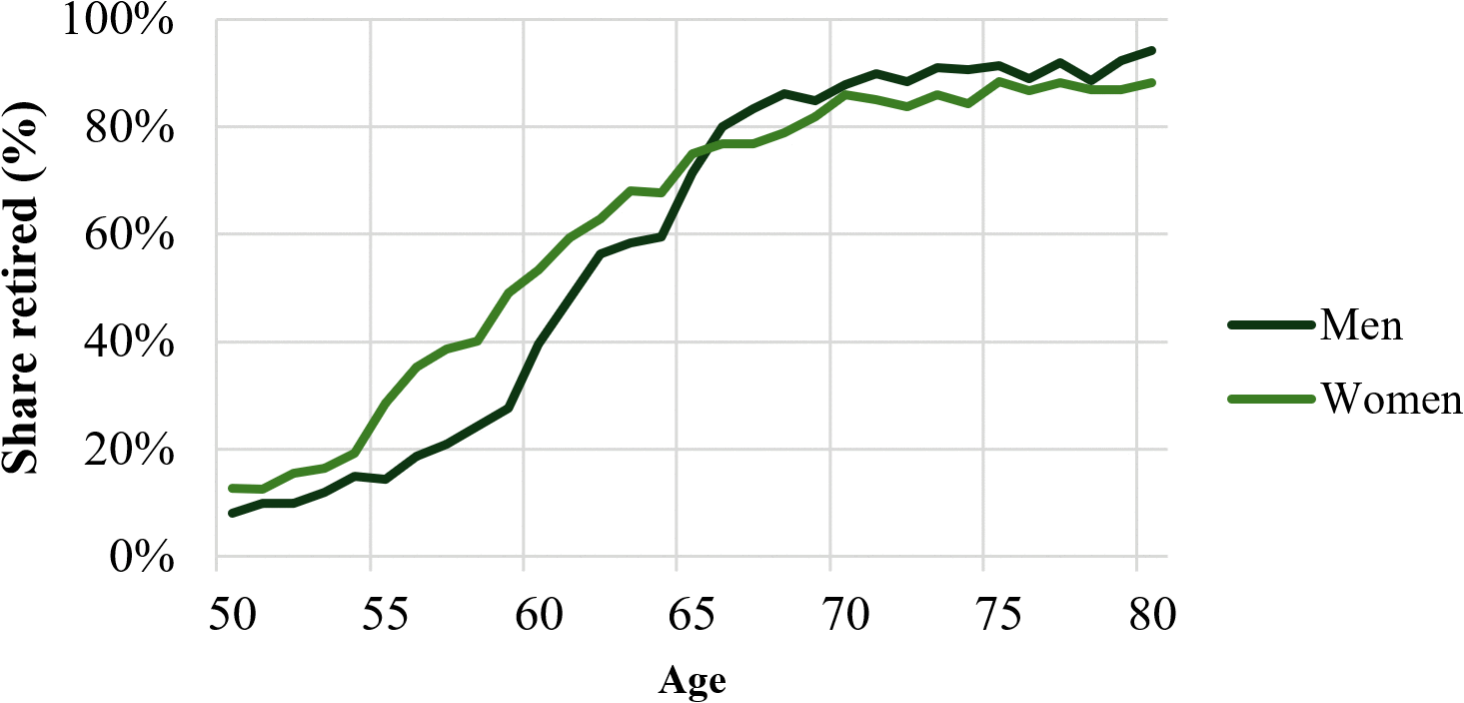
Distribution of retirement by age and sex (PNS 2013 and 2019).

Fig 2 displays the distribution of retirement by age for men and women using pooled data from the 2013 and 2019 PNS. The figure provides a clear picture of how retirement behaviour in Brazil is concentrated around the statutory minimum retirement ages. Retirement rates increase sharply at age 60 for women and age 65 for men, while a smaller but meaningful share of individuals retire before or after these thresholds. This pattern indicates that institutional rules play a central role in shaping retirement decisions, although they do not entirely determine them.

The observed pattern reinforces the validity of our identification strategy. The sharp increase in retirement rates at the statutory ages demonstrates that the minimum legal retirement age is a strong and relevant instrument for retirement status. At the same time, the dispersion of retirements around these ages ensures sufficient variation for identification, supporting the robustness of the instrumental variable approach adopted in this study.

### Regression results: probit and IV probit

Our regression results in Table 4 reveal that retirement is associated with a lower probability of engaging in heavy alcohol use (β = −0.153, *p* < 0.01), cigarette smoking (β = −0.074, *p* < 0.01), while it is associated with an increased probability of using sleep medication (β = 0.230, *p* < 0.01). Moreover, higher income levels correspond to an elevated probability of alcohol consumption but a reduced likelihood of smoking. Similarly, higher levels of education are associated with an increased likelihood of alcohol consumption and a decreased likelihood of using sleep medication and smoking.

Moreover, our analysis indicates that males show a higher propensity for alcohol consumption and cigarette smoking but are less inclined towards using sleep medication. Of interest is that married individuals demonstrate a diminished inclination towards alcohol and tobacco consumption. Conversely, non-white individuals exhibit a higher tendency towards smoking and a lower likelihood of using sleep medication. Furthermore, religious attendance is associated with a reduced probability of alcohol consumption and smoking. All of these associations are statistically significant.

Upon reviewing Table 5, we delve into the outcomes of our IV probit regression, which is employed to tackle endogeneity concerns. Our instrumental variable passed the pertinent statistical tests for weak instruments, thereby indicating the absence of the weak instrument problem. Our findings reveal that retirement enhances the likelihood of embracing physical exercise (β = 0.393, *p* < 0.05) and maintaining a nutritious diet (β = 0.371, *p* < 0.05). Moreover, advancing age has a nonlinear effect on physical exercise: while the linear term suggests a positive association, the negative coefficient on the squared term indicates that this effect diminishes as an individual ages. Elevated income levels also correlate positively with engaging in physical activity and sustaining a healthy dietary pattern.

**Table 5:** IV Probit regressions.

	Heavy Drinker	Smokers	Physical Exercise	Sleep medication	Healthy Diet
	*β (SE)*	*β (SE)*	*β (SE)*	*β (SE)*	*β (SE)*
Retirement	−0.462*	−0.585***	0.393**	0.105	0.371**
	(0.274)	(0.213)	(0.192)	(0.219)	(0.172)
Age	0.052**	0.143***	0.035*	−0.015	0.002
	(0.025)	(0.021)	(0.020)	(0.024)	(0.019)
Age Squared	−0.001***	−0.001***	−0.000***	0.000	−0.000
	(0.000)	(0.000)	(0.000)	(0.000)	(0.000)
Medium income	0.129***	−0.010	0.159***	−0.006	0.255***
	(0.022)	(0.019)	(0.018)	(0.018)	(0.016)
High income	0.211***	−0.068**	0.440***	0.043*	0.512***
	(0.033)	(0.028)	(0.023)	(0.026)	(0.021)
Educ (9–11 years)	0.103***	0.042	0.232***	−0.056**	0.196***
	(0.031)	(0.026)	(0.022)	(0.025)	(0.020)
Educ (12 years)	0.193***	−0.053**	0.356***	−0.116***	0.307***
	(0.026)	(0.022)	(0.019)	(0.022)	(0.017)
Educ (13 + years)	0.077**	−0.146***	0.602***	−0.110***	0.448***
	(0.033)	(0.029)	(0.022)	(0.026)	(0.021)
Biological sex (male)	0.764***	0.131***	0.076***	−0.469***	−0.277***
	(0.042)	(0.029)	(0.023)	(0.024)	(0.023)
Married	−0.218***	−0.281***	0.063***	0.007	0.102***
	(0.019)	(0.016)	(0.015)	(0.016)	(0.013)
Non-white	0.141***	0.044***	−0.021	−0.119***	−0.112***
	(0.021)	(0.017)	(0.015)	(0.016)	(0.013)
Religious attendance	−0.448***	−0.413***	0.244***	−0.010	0.188***
	(0.022)	(0.019)	(0.014)	(0.015)	(0.012)
Rural resident	−0.192***	−0.270***	−0.386***	−0.125***	−0.229***
	(0.029)	(0.026)	(0.021)	(0.022)	(0.017)
North resident	−0.011	−0.125***	−0.156***	−0.297***	−0.123***
	(0.031)	(0.028)	(0.025)	(0.028)	(0.023)
Southeast resident	−0.218***	0.231***	−0.122***	0.109***	0.199***
	(0.026)	(0.024)	(0.019)	(0.021)	(0.016)
South resident	−0.529***	0.322***	−0.141***	0.130***	0.279***
	(0.037)	(0.026)	(0.022)	(0.023)	(0.020)
Midwest resident	−0.174***	0.100***	−0.033	−0.028	0.121***
	(0.035)	(0.031)	(0.026)	(0.029)	(0.023)
2013	−0.254***	0.146***	−0.255***	−0.062***	−0.508***
	(0.021)	(0.016)	(0.014)	(0.015)	(0.013)
Constant	−2.414***	−5.243***	−1.839**	−0.529	−0.823
	(0.840)	(0.704)	(0.719)	(0.824)	(0.659)
Exogeneity test	0.26	0.02	0.18	0.56	0.04
Endogeneity?	No	Yes	No	No	Yes
Kleibergen-Paap	356.80	356.80	356.80	356.80	356.80
Observations	54,741	54,741	54,741	54,741	54,741

* p < 0.1, ** p < 0.05, *** p < 0.01.

Notes: The ‘Exogeneity test’ reports the p-value from the Wald test of exogeneity or a robust Hausman-type test. A p-value below 0.10 indicates evidence of endogeneity, justifying instrumental variable estimation; a p-value above 0.10 suggests no evidence of endogeneity, allowing the variable to be treated as exogenous.

Similarly, higher educational attainment aligns with a greater propensity for adopting these two health-promoting behaviours. Concerning biological sex, our regressions suggest that men exhibit a higher inclination towards physical activity in retirement but a lower tendency towards maintaining a healthy diet. Furthermore, marital status emerges as a positive influencer on physical activity engagement and dietary habits, thereby mirroring the effects observed for religious attendance.

To delve deeper into potential disparities in how retirement impacts individuals based on biological sex, Table 6 presents the outcomes of probit and IV probit regressions, with the sample stratified by biological sex. Our analysis uncovers notable differences warranting attention. Firstly, there is evidence to suggest that retirement diminishes the likelihood of alcohol consumption among men and women in both the probit model (β = −0.137, *p* < 0.01), (β = −0.180, *p* < 0.01). However, this effect lacks statistical significance in the IV probit model. Secondly, retirement is associated with an increased probability of adopting a healthier diet among women in the IV probit model (β = 0.456, *p* < 0.05). This effect, however, is not statistically significant for men in either the probit or IV probit models. In addition, we find that retirement reduces the likelihood of smoking for men (β = −0.488, *p* < 0.10) and women (β = −0.096, *p* < 0.01).

**Table 6:** Probit and IV probit regression by biological sex.

	Drinkers	Smokers	Physical Exercise	Sleep medication	Healthy Diet
Panel A: Men	*β (SE)*	*β (SE)*	*β (SE)*	*β (SE)*	*β (SE)*
Retirement (Probit)	−0.137***	−0.003	0.191***	0.3896***	0.009
	(0.021)	(0.027)	(0.022)	(0.031)	(0.022)
Retirement (IV)	−0.389	−0.488*	0.638***	0.194	0.255
	(0.210)	(0.266)	(0.234)	(0.307)	(0.219)
Exogeneity test	0.38	0.09	0.06	0.53	0.26
Endogeneity?	No	Yes	Yes	No	No
Kleibergen-Paap	248.96	248.96	248.96	248.96	248.96
	**Drinkers**	**Smokers**	**Physical Exercise**	**Sleep medication**	**Healthy Diet**
**Panel B: Women**	***β (SE)***	***β (SE)***	***β (SE)***	***β (SE)***	***β (SE)***
Retirement (Probit)	−0.180***	−0.096***	0.094***	0.154***	0.023
	(0.022)	(0.026)	(0.021)	(0.021)	(0.019)
Retirement (IV)	−0.51	0.334	0.635***	−0.065	0.456**
	(0.627)	(0.284)	(0.215)	(0.230)	(0.195)
Exogeneity test	0.51	0.51	0.80	0.39	0.28
Endogeneity?	No	No	No	No	No
Kleibergen-Paap	42.71	42.71	42.71	42.71	42.71

* p < 0.1, ** p < 0.05, *** p < 0.01.

Notes: The ‘Exogeneity test’ reports the p-value from the Wald test of exogeneity or a robust Hausman-type test. A p-value below 0.10 indicates evidence of endogeneity, justifying instrumental variable estimation; a p-value above 0.10 suggests no evidence of endogeneity, allowing the variable to be treated as exogenous.

### Regression results: OLS and IV OLS

To evaluate the robustness of our probit models, Tables 7 and 8 report the results from our OLS and IV OLS regressions with our ‘continuous’ health behaviour dependent variables. Endogeneity was only detected in our physical exercise model. Reviewing the OLS regression outcomes in Table 7, we find that retirement is associated with a decrease in the quantity of alcohol consumption (β = −0.241; p < 0.01) and smoking (β = −0.155; p < 0.01), and with an increase in the weekly minutes of participation in physical exercise (β = 14.00; p < 0.01) and in the number of days using sleep medication (β = 0. 512; p < 0.01).

**Table 7:** OLS regressions.

	Drinks	Cigarettes Smoked	Physical Exercise	Sleep medication	Healthy Diet
	*β (SE)*	*β (SE)*	*β (SE)*	*β (SE)*	*β (SE)*
Retirement	−0.241***	−0.155***	14.005***	0.512***	0.017
	(0.022)	(0.051)	(1.627)	(0.040)	(0.038)
Age	−0.082***	0.180***	3.875***	−0.070**	0.076**
	(0.020)	(0.039)	(1.170)	(0.030)	(0.030)
Age Squared	0.000**	−0.002***	−0.039***	0.001**	−0.000
	(0.000)	(0.000)	(0.009)	(0.000)	(0.000)
Medium income	0.231***	−0.001	14.050***	−0.006	0.980***
	(0.024)	(0.051)	(1.401)	(0.036)	(0.037)
High income	0.511***	−0.116	44.961***	0.055	1.761***
	(0.040)	(0.087)	(2.547)	(0.057)	(0.056)
Educ (9–11 years)	0.170***	0.217***	16.373***	−0.125**	0.759***
	(0.037)	(0.082)	(2.088)	(0.052)	(0.054)
Educ (12 years)	0.252***	−0.058	32.876***	−0.269***	1.174***
	(0.032)	(0.068)	(2.059)	(0.045)	(0.046)
Educ (13 + years)	0.184***	−0.290***	62.401***	−0.301***	1.586***
	(0.037)	(0.086)	(2.609)	(0.056)	(0.055)
Biological sex (male)	1.117***	0.689***	11.435***	−0.831***	−1.122***
	(0.023)	(0.047)	(1.389)	(0.032)	(0.034)
Married	−0.321***	−0.587***	3.125**	−0.030	0.433***
	(0.022)	(0.045)	(1.324)	(0.032)	(0.033)
Non-white	0.097***	−0.037	−0.109	−0.261***	−0.354***
	(0.022)	(0.049)	(1.459)	(0.035)	(0.035)
Religious attendance	−0.546***	−0.988***	22.097***	−0.037	0.665***
	(0.019)	(0.041)	(1.357)	(0.033)	(0.033)
Rural resident	−0.231***	−0.631***	−27.879***	−0.140***	−0.805***
	(0.027)	(0.053)	(1.337)	(0.038)	(0.042)
North resident	−0.095**	−0.156***	−13.924***	−0.451***	−0.701***
	(0.038)	(0.053)	(1.758)	(0.037)	(0.048)
Southeast resident	−0.193***	0.788***	−7.087***	0.357***	0.714***
	(0.028)	(0.062)	(1.769)	(0.045)	(0.044)
South resident	−0.425***	0.949***	−6.338***	0.364***	0.898***
	(0.030)	(0.081)	(2.314)	(0.057)	(0.053)
Midwest resident	−0.187***	0.415***	−3.169	0.033	0.336***
	(0.036)	(0.073)	(2.241)	(0.052)	(0.054)
2013	−0.411***	0.351***	−21.890***	−0.081**	−1.458***
	(0.022)	(0.048)	(1.320)	(0.033)	(0.033)
Constant	4.728***	−2.703**	−50.899	3.872***	4.618***
	(0.640)	(1.258)	(36.986)	(0.947)	(0.959)
R-squared	0.11	0.04	0.08	0.03	0.20
Exogeneity test	0.73	0.029	0.33	0.34	0.39
Endogeneity?	No	Yes	No	No	No
Observations	54,741	54,741	54,741	54,741	54,741

* p < 0.1, ** p < 0.05, *** p < 0.01.

Notes: The ‘Exogeneity test’ reports the p-value from the Wald test of exogeneity or a robust Hausman-type test. A p-value below 0.10 indicates evidence of endogeneity, justifying instrumental variable estimation; a p-value above 0.10 suggests no evidence of endogeneity, allowing the variable to be treated as exogenous. Definitions and measurement units for all continuous variables (drinks, cigarettes smoked, minutes of physical activity, and days using sleep medication) are provided in Table 2.

**Table 8:** IV OLS regressions.

	Drinks	Cigarettes Smoked	Physical Exercise	Sleep medication	Healthy Diet
	*β (SE)*	*β (SE)*	*β (SE)*	*β (SE)*	*β (SE)*
Retirement	−0.347	−1.600**	33.226*	0.080	0.420
	(0.311)	(0.677)	(19.848)	(0.456)	(0.471)
Age	−0.073*	0.298***	2.314	−0.035	0.043
	(0.038)	(0.075)	(2.119)	(0.044)	(0.049)
Age Squared	0.000	−0.002***	−0.031**	0.000	−0.000
	(0.000)	(0.000)	(0.013)	(0.000)	(0.000)
Medium income	0.235***	0.048	13.402***	0.008	0.967***
	(0.026)	(0.057)	(1.577)	(0.039)	(0.040)
High income	0.511***	−0.118	44.985***	0.054	1.761***
	(0.040)	(0.088)	(2.546)	(0.057)	(0.056)
Educ (9–11 years)	0.166***	0.173**	16.961***	−0.138**	0.771***
	(0.039)	(0.084)	(2.188)	(0.054)	(0.056)
Educ (12 years)	0.250***	−0.080	33.165***	−0.275***	1.180***
	(0.033)	(0.069)	(2.082)	(0.046)	(0.047)
Educ (13 + years)	0.183***	−0.306***	62.620***	−0.306***	1.590***
	(0.037)	(0.087)	(2.622)	(0.057)	(0.056)
Biological sex (male)	1.107***	0.554***	13.244***	−0.871***	−1.084***
	(0.037)	(0.077)	(2.238)	(0.053)	(0.055)
Married	−0.324***	−0.629***	3.688**	−0.043	0.445***
	(0.023)	(0.049)	(1.437)	(0.035)	(0.035)
Non-white	0.097***	−0.031	−0.196	−0.259***	−0.356***
	(0.022)	(0.050)	(1.469)	(0.036)	(0.035)
Religious attendance	−0.546***	−0.985***	22.054***	−0.036	0.664***
	(0.019)	(0.041)	(1.356)	(0.033)	(0.033)
Rural resident	−0.226***	−0.564***	−28.778***	−0.120***	−0.824***
	(0.031)	(0.062)	(1.583)	(0.043)	(0.047)
North resident	−0.102**	−0.253***	−12.641***	−0.480***	−0.674***
	(0.043)	(0.071)	(2.224)	(0.048)	(0.057)
Southeast resident	−0.196***	0.742***	−6.473***	0.343***	0.727***
	(0.030)	(0.066)	(1.875)	(0.048)	(0.046)
South resident	−0.424***	0.960***	−6.474***	0.367***	0.895***
	(0.030)	(0.082)	(2.334)	(0.058)	(0.054)
Midwest resident	−0.194***	0.319***	−1.894	0.005	0.363***
	(0.041)	(0.086)	(2.572)	(0.061)	(0.063)
2013	−0.411***	0.364***	−22.058***	−0.078**	−1.461***
	(0.022)	(0.049)	(1.348)	(0.033)	(0.033)
Constant	4.409***	−7.058***	7.033	2.570*	5.833***
	(1.350)	(2.636)	(74.496)	(1.526)	(1.729)
Exogeneity test	0.73	0.029	0.33	0.34	0.39
Endogeneity?	No	Yes	No	No	No
Kleibergen-Paap	290.83	290.83	290.83	290.83	290.83
Observations	54,741	54,741	54,741	54,741	54,741

* p < 0.1, ** p < 0.05, *** p < 0.01.

Notes: The ‘Exogeneity test’ reports the p-value from the Wald test of exogeneity or a robust Hausman-type test. A p-value below 0.10 indicates evidence of endogeneity, justifying instrumental variable estimation; a p-value above 0.10 suggests no evidence of endogeneity, allowing the variable to be treated as exogenous. Definitions and measurement units for all continuous variables (drinks, cigarettes smoked, minutes of physical activity, and days using sleep medication) are provided in Table 3.

Our IV OLS results in Table 8 suggest a negative, though statistically insignificant, association between retirement and alcohol consumption (β = −0.347; *p* < 0.01), while retirement has a marginally significant positive effect on participation in physical activity (β = 33.22; p < 0.10). These results are broadly consistent with the patterns observed in our probit analysis.

### Regression results: stratified by sex

Table 9 presents marginal effects from probit models and coefficient estimates from OLS models for each health behaviour outcome, stratified by all individuals (Panel A), men (Panel B), and women (Panel C). Our analysis reveals a statistically significant negative association between retirement and alcohol consumption overall (Panel A). Moreover, retirement is associated with a reduction in the quantity of alcohol consumed, with this effect being particularly pronounced among women (Panel C) compared to men (Panel B). With respect to smoking behaviour, our findings suggest that retirement is linked to a modest 1% decrease in the likelihood of being a smoker and a reduction of 0.15 cigarettes smoked per day (Panel A). Notably, this effect is more pronounced among men (Panel B), with retirement being associated with a 9% decrease in the likelihood of smoking and a substantial reduction of 1.62 cigarettes smoked daily.

**Table 9:** Marginal effects and OLS results.

	Heavy Drinker	Smokers	Physical Exercise	Sleep medication	Healthy Diet
**Panel A: Total**	***β***	***β***	***β***	***β***	***β***
Probit	−0.18***	−0.01***	0.03***	0.04***	0.006
IV Probit	−0.05*	−0.10***	0.10**	0.004	0.12***
Continuous (OLS)	−0.241***	−0.155***	14.00***	0.512***	0.017
Continuous (IV)	−0.347	−1.600**	33.22***	0.080	0.420
Endogeneity	No	Yes	No	No	No
	**Heavy** **Drinker**	**Smokers**	**Physical Exercise**	**Sleep medication**	**Healthy Diet**
**Panel B: Men**	***β***	***β***	***β***	***β***	***β***
Probit	−0.025***	−0.005	0.04***	0.05***	0.002
IV Probit	−0.07	−0.09*	0.16**	0.02	0.07
Continuous (OLS)	−0.337***	−0.149*	19.83***	0.597***	−0.004
Continuous (IV)	−0.703	−1.622*	38.44	−0.037	0.256
Endogeneity	No	Yes	Yes	No	No
	**Drinkers**	**Smokers**	**Physical Exercise**	**Sleep medication**	**Healthy Diet**
**Panel C: Women**	***β***	***β***	***β***	***β***	***β***
Probit	−0.09***	−0.01***	0.02***	0.04***	0.009
IV Probit	−0.03	−0.07	0.06	−0.08	0.19
Continuous (OLS)	−0.143***	−0.130**	2.048***	0.467***	0.032
Continuous (IV)	0.205	−0.098	12.832	−0.935	0.648
Endogeneity	No	No	No	No	No

* p < 0.1, ** p < 0.05, *** p < 0.01.

Note: Probit results are reported as marginal effects evaluated at the sample means.

The most notable impact emerges with regard to physical exercise, where retirement is associated with a 3% increase in the likelihood of engaging in physical activity, coupled with an average extension of 14.00 minutes of physical exercise per week (Panel A). This effect is particularly pronounced among men (Panel B), with retirement leading to a 15% increase in the likelihood of participating in physical exercise for men. Finally, the data show that retirement in Brazil is linked to a 4% increase in the likelihood of using sleep medication, with this effect consistently being observed across both biological sexes. Furthermore, retirement shows a propensity to bolster the likelihood of maintaining a healthy diet by 12%, although the results from the stratified sample do not achieve statistical significance.

It is important to note that our IV estimates differ from the OLS and probit results. These differences are expected and can be attributed to the presence of endogeneity in the retirement variable. Specifically, individuals who retire early may do so because of unobserved health problems or lifestyle preferences, which can lead to biased estimates in non-IV models. The IV approach addresses this issue by isolating exogenous variation in retirement status based on the statutory minimum retirement age, thereby identifying the Local Average Treatment Effect (LATE). This estimate reflects the causal impact of retirement for individuals whose decision to retire was induced by reaching the eligibility threshold, rather than by unobserved personal factors. As a result, the IV results should be interpreted as a more reliable measure of the causal effect of retirement, particularly among those individuals whose retirement decisions are directly influenced by the statutory eligibility rules – i.e., those who retire because they reach the minimum legal retirement age, rather than for personal or health-related reasons. This group represents a key policy-relevant population for evaluating the impact of retirement regulations. This aligns with prior research noting that retirement decisions are often influenced by unobserved factors, such as latent health conditions or personal preferences, which can bias non-IV estimates downward [7,18].

Beyond identification concerns, it is also important to consider the practical significance of our estimated effects. While a 13-minute increase in weekly physical activity may appear modest at the individual level, such changes are meaningful when viewed from a population health perspective, particularly in the context of Brazil’s large and ageing population. These effects align with plausible mechanisms linking retirement to healthier behaviours, including reduced stress, greater discretionary time, and improvements in mental well-being following labour market exit [57,58]. Enhanced mental health at retirement may also lessen reliance on maladaptive coping strategies such as smoking or excessive alcohol consumption [58], while women’s stronger social networks may provide additional support for adopting healthier lifestyles. Collectively, these pathways suggest that even relatively small behavioural shifts associated with retirement can have significant implications for promoting healthy ageing at the population level [7].

## Discussion

The outcomes from the data analysis above highlight two pivotal observations: firstly, retirement is markedly linked with positive shifts in health behaviour, and, secondly, a spectrum of economic and social factors has substantial influence over the various aspects that make up health behaviour. The sex-stratified results provide further insights into how retirement may affect men and women differently. These differences likely reflect gendered patterns in social roles, social networks, and labour market attachment.

Women, for instance, often maintain stronger social support networks, which may facilitate healthier lifestyle changes, such as improved diet and physical activity, once they retire [58]. Men’s behavioural changes, by contrast, may be more closely tied to reductions in work-related stress and increased discretionary time following retirement. Furthermore, women’s longer life expectancy and greater engagement with preventive healthcare may provide more opportunities to adopt healthier behaviours after leaving the workforce. Taken together, these findings underscore the importance of considering gender-specific pathways when examining the relationship between retirement and health behaviours [57].

Table 10 provides a comprehensive overview of the pertinent findings in the literature and thus serves as an invaluable resource for contextualising our results. Notably, the relationship between retirement and health behaviours shows considerable variability across the studies we have adduced. For instance, for alcohol consumption, some researchers report a positive correlation between retirement and health behaviours [19,33,60,61,64] while others find no statistically significant association [25,28,31,65,66].

**Table 10:** Studies on the causal impact of retirement on health behaviours.

Authors	Country	Method	Results
[2] Dave, Rashad and Spasojevic (2008)	USA	FE	PA (-)
[3] Insler (2014)	USA	FE-IV	S (-), PA (+)
[6] Gorry and Slavov (2023)	USA	IV	D (0), S (+), SD (+), HD (0), PA (+)
[15] Motegi, Nishimura and Oikawa (2020)	USA, JP, 5 EU	FE-IV	S (0), D (+), PA (+)
[19] Eibich (2015)	Germany	RDD	S (-), D (+), SD (+), HD (0)
[22] Chen, Wakabayashi and Yuda (2024)	Japan	FE-IV	PA (+), D (0), S (0)
[25] Zhu (2016)	Australia	FE-IV	S (-), D (0), PA (+)
[27] Behncke (2012)	11 EU	IV	PA (-)
[28] Rose (2020)	England	RDD, FE-IV	S (0), D (0), PA (0), SD (+)
[56] Neuman (2008)	USA	IV	PA (0)
[57] Kämpfen and Maurer (2016)	USA	IV	PA (+)
[60] Celidoni and Rebba (2017)	Europe	FE-IV	PA (+), S (0), D (+)
[62] Binh Tran and Zikos (2019)	Australia	FE-IV	S (0), D (0), PA (+)
[61] Müller and Shaikh (2018)	19 EU	RDD	PA (+), D (+), S (0)
[64] Feng, Li and Smith (2020)	China	RDD	PA (0), S (0), D (+)
[66] Che and Li (2018)	China	IV	PA (+), S (0), D (0)
[65] Henkens Solinge and Gallo (2008)	Netherlands	ML	PA (+), S (0), D (0)
[31] Oshio and Kan (2017)	Japan	FE-IV	S (-), D (0), PA (+)
[68] Eyjólfsdóttir et al. (2019)	Sweden	PSM	PA (0)
[69] Grøtting and Lillebø (2020)	Norway	RDD	PA (+)
[70] Fé and Hollingsworth (2016)	UK	RDD	SD (+)
[71] Ding et al. (2016)	Australia	Logit	S (-), D (0), SD (+), HD (0) PA (+)

**Notes:** 0 = no effect of retirement on the dependent variable, + = positive effect of retirement on the dependent variable, and - = negative effect of retirement on the dependent variable (p < 0.05). The plus and minus signs represent the empirical direction found in the respective study. Identification Strategy: PSM = Propensity score matching; IV = Instrumental Variables; FE = Fixed effects; RDD = Regression discontinuity design; ML = Multinomial logit. Results: SD = Sleep duration; D = Drinking; S = Smoking; PA = Physical activity; HD = Healthy diet.

Although the literature frequently emphasises the potential benefits of retirement for health-related behaviours, the relationship is theoretically ambiguous. Some studies report improvements in lifestyle, while others document increased sedentary behaviour, greater alcohol use, or social isolation. This mixed evidence highlights the importance of empirically testing each behaviour individually, as is summarised in Table 10.

In contrast with what much of the literature suggests, our findings indicate that retired individuals are more inclined to curb their alcohol consumption. One potential explanation for this disparity may lie in workplace environmental factors, which could foster alcohol consumption as a means to cope with work-related anxiety or stress [67]. However, akin to findings from other investigations, our results support the notion that men generally reduce their use of alcohol when they retire, meaning that, with two men of the same age, the one who is still working will likely consume more alcohol than his retired counterpart.

Smoking represents another critical variable owing to its profound health ramifications, notably including its links to lung cancer and cardiovascular disease, as well as a variety of other chronic conditions. The influence of retirement on smoking behaviour yields mixed findings within the literature. While some studies have failed to discern any statistically significant relationship between smoking and retirement [13,28,60,64,66], others have reported a negative association [3,25]. Our findings also indicate a negative relationship between retirement and smoking behaviour, thereby signifying a reduction in the likelihood of cigarette smoking post-retirement. Nevertheless, we observe a modest impact within this relationship, particularly among women, a trend akin to the findings reported by [31].

The impact of retirement on physical activity is varied. Some authors have identified that retirement reduces the amount of physical activity [2,27], while others have reported no statistically significant relationship [28,61,64,65,67,68]. However, most studies have found that retirement increases the level of physical activity [6,25,31,60,65–67,69]. This latter outcome is aligned with our findings, which indicate that retirement increases the likelihood of engaging in physical activity in Brazil. This could be attributed to the increased availability of time for activities such as physical exercise post-retirement and a desire to improve personal well-being to prolong one’s life and overall enjoyment of retirement [15].

With regard to sleep medication, the relevant literature often relies on sleep duration as a key variable, indicating the amount of time devoted to rest. Previous studies suggest that individuals increase their sleeping time post-retirement [6,20,28,70,71]. However, our study adopted a distinct metric for analysing sleep patterns, and our findings do not present compelling evidence of a statistically significant association between retirement and the use of sleep medication. Importantly, unlike most studies that focus on sleep duration or quality, our analysis uses sleep medication as an outcome. This represents a novel contribution, especially in a context where pharmacological sleep modification is becoming increasingly common among older adults. Importantly, this association is adjusted for both age and age squared, which account for linear and accelerating effects of ageing, ensuring that the observed increase in sleep medication use among retirees reflects retirement-related changes rather than simple age differences.

Concerning dietary habits, some authors have reported a lack of statistically significant association between retirement and adhering to a healthy diet [6,16]. That said, a recent study using U.S. datasets points out that retirees, particularly men, change their former patterns of eating out frequently and devote more time to (often healthier) food preparation at home [6]. Consistent with these findings, our study also reveals limited evidence to support a positive relationship between retirement and adopting a healthy diet.

Overall, our results suggest that retirement significantly influences supporting healthy behaviours in Brazil. Moreover, a consistent pattern emerges whereby all coefficients have increased magnitudes in instrumental variable regression, which is akin to the other findings [62]. This phenomenon may stem from reverse causality, such as where individuals with poorer health behaviours tend to retire earlier than those with healthier habits. As a consequence, the estimated effect of retirement is vulnerable to attenuation bias. Naturally, the magnitudes of the estimates tend to escalate once we address the endogeneity of retirement through our instrumental variable approach, which aligns with the theoretical framework proposed in previous research [62]. In addition, we conducted regressions utilising various age ranges (e.g., from 50 years to 100 years) and included age squared as an additional independent variable (results available upon request). However, this had minimal impact on our findings.

It is important to consider that changes in health behaviours observed around the statutory retirement age could be influenced not only by the receipt of pension income, but also by broader social norms that associate the ages of 60 or 65 with the socially expected timing of labour market exit. In this sense, our estimates capture the combined effect of institutional and normative retirement, both of which represent meaningful transitions out of the workforce. This interpretation aligns with previous literature that identifies the statutory retirement age as not only a legal threshold but also a normative reference embedded in biographical expectations and reinforced by social conventions regarding the appropriate timing of retirement [13].

These results are essential in the context of many developed economies seeking to extend the working life of their citizens, something which has been accomplished primarily by raising the compulsory age thresholds for accessing state-funded pensions [25]. This trend has also been observed in developing nations such as Brazil. For instance, Brazil has undergone seven pension reforms since 1993 to tighten access to social security in the face of increased pressures on government expenditure [72]. In this context, delaying retirement in Brazil has the clear potential to adversely affect positive health behaviours, thereby leading to a potentially greater quantum of costly health issues that would increase the burden on the public health system.

While this investigation yields significant findings, such as the above, it is subject to limitations, including the possibility that the individual’s job status before retirement could influence their health behaviours post-retirement [73]. It is also worth noting that the statutory retirement ages for men (65) and women (60) remained unchanged during our study period; however, the 2019 pension reform, which raised the minimum age for women to 62, presents an opportunity for future research to exploit this policy change for stronger identification. A potential limitation of this study is the possibility of anticipatory behavioural changes prior to retirement, which could slightly attenuate the estimated effects. Evidence of such pre-retirement adjustments has been reported in other contexts [16], but their magnitude in Brazil is likely modest. Testing these mechanisms would require longitudinal data with repeated pre-retirement observations, which is beyond the scope of the cross-sectional PNS data. However, our study is significant for several reasons. Firstly, as far as we know, it presents the first findings on the impact of retirement on health behaviours in Brazil. Secondly, we addressed the issue of endogeneity associated with retirement and utilised various metrics of healthy behaviours by employing large and representative samples in Brazil. These methodological approaches offer valuable insights into understanding the effects of retirement on health behaviour in the Brazilian context, with the possibility of these insights holding true in similar national and economic settings around the globe.

## Conclusion

This study has explored the relationship between retirement and health behaviours within Brazil, a developing nation where the average life expectancy has increased in recent years. Using data from the 2013 and 2019 Brazilian National Health Surveys (PNS), we aimed to contribute to the existing literature on retirement and health behaviours by presenting, to the best of our knowledge, the first empirical evidence on the impact of retirement on health behaviour.

The overall results support an array of work mainly carried out so far in developed countries, especially with regard to retirement, having a positive relationship with certain health-related variables, such as reducing the consumption of cigarettes and improving physical exercise and dietary outcomes. These outcomes suggest that policymakers in Brazil and cognate jurisdictions need to think carefully about the degree to which putting upward pressure on retirement ages, such as increasing the minimum age to access state pensions, could have adverse long-term effects both with respect to the health of retired citizens and the public purse. In short, increasing policy-determined retirement thresholds could result in greater burdens from a public health perspective than would be the case if workers were permitted to retire earlier and then invest in and work on their own physical and mental well-being.
